# A Ferroptosis-Related Gene Model Predicts Prognosis and Immune Microenvironment for Cutaneous Melanoma

**DOI:** 10.3389/fgene.2021.697043

**Published:** 2021-08-10

**Authors:** Congcong Xu, Hao Chen

**Affiliations:** Hospital of Dermatology, Chinese Academy of Medical Sciences and Peking Union Medical College, Nanjing, China

**Keywords:** cutaneous melanoma, ferroptosis, immune, prognosis, gene model

## Abstract

**Background:**

Cutaneous melanoma is a common but aggressive tumor. Ferroptosis is a recently discovered cell death with important roles in tumor biology. Nevertheless, the prognostic power of ferroptosis-linked genes remained unclear in cutaneous melanoma.

**Methods:**

Cutaneous melanoma patients of TCGA (The Cancer Genome Atlas) were taken as the training cohort while GSE65904 and GSE22153 as the validation cohorts. Multifactor Cox regression model was used to build a prognostic model, and the performance of the model was assessed. Functional enrichment and immune infiltration analysis were used to clarify the mechanisms.

**Results:**

A five ferroptosis-linked gene predictive model was developed. ALOX5 and GCH1 were illustrated as independent predictive factors. Functional assessment showed enriched immune-linked cascades. Immune infiltrating analysis exhibited the distinct immune microenvironment.

**Conclusion:**

Herein, a novel ferroptosis-related gene prognostic model was built in cutaneous melanoma. This model could be used for prognostic prediction, and maybe helpful for the targeted and immunotherapies.

## Introduction

Cutaneous melanoma, an aggressive malignant tumor with increasing incidence, accounts for more than 80% of all skin cancer deaths (Chen et al., 2019; Yumnam et al., 2020). Globally, approximately 232,100 of cutaneous melanoma are newly diagnosed, and 55,500 patients die each year (Schadendorf et al., 2018). Satisfactory therapeutic effects have not been achieved in melanoma. Surgical resection is preferred for early-stage melanoma but may produce long scars and lead to detrimental psychologic effects (Weyers, 2019). Melanoma is prone to recur or metastasize even after the combination of surgery, radiotherapy, and chemotherapy (Huang et al., 2019). Immune checkpoint inhibitors along with molecular-targeted treatment have improved the prognosis of melanoma patients, but most patients do not show long-lasting responses to these therapies (Davis et al., 2019; Wada-Ohno et al., 2019). Poorer survival may be related to multiplex clinical and histopathological features, such as older age, elevated lactate dehydrogenase levels, ulceration, increased thickness of tumor, and higher mitotic rate (Strudel et al., 2020). However, the predictive power of the traditional clinical information is limited. Therefore, novel prognostic biomarkers are strongly needed to improve risk stratification and inform treatment optimization in melanoma patients.

Ferroptosis is a novel type of modulated cell death that is different from other forms of cell death, i.e., apoptosis, autophagic cell death, as well as necrosis. It is typified by iron-dependent lipid peroxide aggregation (Dixon et al., 2012). Shrinkage of the cell volume along with increased density of the mitochondrial membrane are the main morphological features of ferroptosis (Yu et al., 2017). Ferroptosis has a core role in many diseases, e.g., ischemic organ injury, cancer, as well as neurodegeneration (Stockwell et al., 2017). Abnormal activation of neuronal ferroptosis is an important pathogenesis of neurological diseases, and diet that affects ferroptosis can be an adjuvant therapy for these diseases (Mao et al., 2021). Recently, the stimulation of ferroptosis has mushroomed as an alternative therapeutic approach to tumor suppression, especially for therapy-resistant patients (Hassannia et al., 2019; Liang et al., 2019). Ferroptosis exerts effectiveness in radiotherapy-induced cancer suppression and mediates the synergy of radiation therapy and immunotherapy (Lei et al., 2021). Apart from drivers and inducers of ferroptosis, many genes have been defined as suppressors, inhibitors, markers, and ferroptosis–disease associations (Zhou and Bao, 2020). Ferroptosis may functionally act as a double-edged sword that can either promote or inhibit tumor progression processes in different environments (Jiang et al., 2020). Ferroptotic cancer cells stimulate or suppress tumor immunity by many steps (Cheng et al., 2020). The lymphatic environment can protect melanoma cells from ferroptosis in the blood (Ubellacker et al., 2020). By mining public databases, the effective ferroptosis-related gene prognostic signatures were developed in hepatocellular carcinoma and glioma (Liang et al., 2020; Liu et al., 2020; Tang et al., 2020). The risk score based on ferroptosis-related gene signature can also predict glioma immunotherapy (Wan et al., 2021). Nevertheless, the relationship of ferroptosis-linked genes with the prognosis of cutaneous melanoma patients is still largely unknown.

Herein, we first explored the relative expression of ferroptosis-associated genes in cutaneous melanoma samples. Besides, we created and verified a prognostic model with ferroptosis-linked genes. Further functional enrichment and immune infiltration analysis were performed to elucidate the responsible mechanisms.

## Materials and Methods

### Data Collection and Preprocessing

Date of gene expression and matching clinical data for human cutaneous melanoma (SKCM) tumors were abstracted from TCGA (The Cancer Genome Atlas) program. FPKM (Fragments Per Kilobase of transcript per Million mapped reads) represented the expression values for all genes. This dataset consisted of 482 samples, comprising 471 tumor samples and one non-malignant sample. The number of samples containing clinical information was 458. To explore the pattern of gene expression in a logarithmic form, we removed genes with FPKM values of zero across more than 100 samples and added the value of 0.001 to every FPKM value before log2 transformation. For multiple probes corresponding to one gene, the max expression value was taken as the expression value of the gene. Finally, 21,550 gene expression profiles were obtained. The FPKM expression profile of 233 non-sun exposed skin samples from the Genotype-Tissue Expression (GTEx) Project were obtained in order to increase the number of normal patient samples. The batch effect was removed using the removeBatchEffect function from the limma package (v 3.44.3) (Ritchie et al., 2015) to analyze the expression data of TCGA and GTEx data together (Wang et al., 2018).

In addition, we abstracted the GSE65904 and GSE22153 datasets as a validation set. These datasets were embedded in the GPL10558 platform (Illumina HumanHT-12 V4.0 expression beadchip) and, respectively, included gene expression profiles and the survival data of 210 and 54 melanoma samples of the patient. We converted the probes into the corresponding gene symbols on the basis of the annotation information of platform. When multiple probes correspond to the same gene, the max expression value was considered as the gene expression value.

### Determination of Differentially Expressed Genes

Differentially expressed genes (DEGs) between tumor samples and non-malignant tissues were determined by limma package in R, with the criteria of log2 fold change (log2FC) > 1 along with a false discovery rate (FDR) < 0.05. Gene expression levels were normalized, respectively, using the normalizeBetweenArrays function in limma before data analysis, to ensure that the expression distributions of each sample are similar across the overall matrix.

### Gene Ontology, Kyoto Encyclopedia of Genes and Genomes, and Reactome Enrichment Analysis

Gene Ontology (GO) terms along with Kyoto Encyclopedia of Genes and Genomes pathway analyses (KEGG^1^) were employed to interpret the gene set of interest, using the clusterProfiler R package (v 3.16.1) (Yu et al., 2012). The GO analysis reveals the gene function in the biology process, molecular function, and cell component. KEGG is a data resource employed to explore high-level functions, as well as utilities of a distinct biological system at a molecular level. Reactome data resource^2^, an integrated database for signaling pathway enrichment analysis, was also used in the enrichment analysis in the ReactomePA (v 1.32.0) package (Yu and He, 2016).

### Immune Cell Infiltration Abundance Analysis

On the basis of gene expression profiles of 458 cutaneous melanoma samples containing corresponding clinical data in TCGA, the infiltrations of 24 types of immune cells were explored in the samples with Immune Cell Abundance Identifier (ImmuCellAI). The assessed immune cells consisted of 18 T-cell subtypes, B cells, macrophage cells, NK cells, neutrophil cells, dendritic cells (DCs), and monocyte cells. ImmuCellAI is a new gene set signature-based approach that estimates the abundance of 24 kinds of immune cells consisting of 18 T-cell subsets, B cells, monocytes, NK cells, macrophage cells, neutrophil cells, and DCs.

### Consensus Cluster Analysis

According to the expression patterns of ferroptosis-linked DEGs, we performed consensus clustering of 458 cutaneous melanoma samples with the R package “ConsensusClusterPlus” (v 1.52.0) (Wilkerson and Hayes, 2010). The clustering approach was K-means algorithm with Euclidean distance. Consensus clustering was run 1,000 times with all of the genes and 80% of the samples randomly selected on each iteration, and the random seed was 12,621. The cluster numbers k were selected by the elbow approach (Li et al., 2019).

### Construction and Verification of Risk Score Model

We obtained 253 ferroptosis-linked genes from the FerrDb data resource (Zhou and Bao, 2020). The intersection of the 253 genes and DEGs identified 19 differentially expressed ferroptosis-linked genes between non-malignant and cancer samples. Multifactor Cox proportional hazard regression model with stepwise regression was employed to perform multivariate analysis. According to the regression model, we obtained the risk score containing the expression of five genes with the following formula: RiskScore = −0.18811 × ALOX5 − 0.07911 × ANGPTL7 + 0.13678 × TXNIP + 0.28800 × SLC2A6 − 0.46713 × GCH1. On the basis of the median risk score, the samples were stratified into high-risk group and low-risk group (Du et al., 2020), and the Kaplan–Meier curves of the two groups were plotted using the survminer package (v 0.4.8^3^) to compare the survival differences. Subsequently, we constructed the ROC curve to explore the prognostic value in 9, 11, 13, 15, and 20 years by using the survivalROC package (v 1.0.3^4^).

To ensure that GEO and TCGA expression profiles are comparable, we removed the batch effect with the RemoveBatchEffect function in limma package in R. The batch effect between the GSE65904 dataset and the de-batched TCGA data and the batch effect between the GSE22153 dataset and de-batched TCGA data were both removed. On the basis of the expression of five genes in the previous model, multifactor Cox regression assessment was performed to establish a new risk score model. Then, we determined the risk score for every sample, stratified the samples into high- and low-risk groups on the basis of the median value of risk score, and then verified whether the risk score is an independent predictive factor. ROC curves were drawn in 2, 4, 6, 8, and 10 years for the GSE65904 data and in 0.5, 1, 1.5, 2, and 2.5 years for the GSE22153 data.

### Real-Time PCR Analysis

Total RNA was extracted, respectively, from normal human epidermal melanocytes (NHEM) and melanoma cell lines (A375, SK-MEL-28, and MV3) using RNAiso Plus (Takara Biotechnology Co., Ltd., Shija, Japan). cDNA was synthesized from this RNA using HiScript II Q RT SuperMix (R223-01; Vazyme Biotech, Nanjing, China). Real-time PCR was performed on a LightCycler^®^ 480 Instrument II (Roche, Basel, Switzerland) using ChamQ SYBR qPCR Master Mix (Q331-02; Vazyme Biotech, Nanjing, China). The 2^–ΔΔCt^ method was used to calculate the relative gene expression levels and then normalized against GAPDH. The primers are listed in Supplementary Table 1.

### Immunoblot Analysis

Total proteins of NHEM, A375, SK-MEL-28, and MV3 were extracted by RIPA lysis buffer. Supernatant containing proteins was quantified with a BCA kit (Beyotime, Beijing, China). Proteins were separated with SDS-PAGE and transferred to a PVDF membrane. Membranes were blocked with 5% BSA in TBST for 1 h at room temperature and then incubated with primary antibodies overnight at 4°C. After washing, membranes were incubated with matched secondary antibodies for 1 h. Proteins were detected using a chemiluminescence kit.

### Statistical Analyses

Statistical analyses were implemented in R, version 4.0.2. Throughout the study, *p*-values were adjusted using the FDR, and statistical significance was assumed at an adjusted *p*-value threshold of 0.05. The screening criteria for DEGs were FDR < 0.05 and log2FC > 1. Multivariable Cox proportional hazards regression model with stepwise regression was performed to define risk factors, and patients were clustered into high- and low-risk groups on the basis of the risk scores. The survival probabilities between high- and low-risk groups were assessed by the Kaplan–Meier curve, and the difference in survival rate was compared by the log-rank test. We performed ROC curves and calculated the AUC to assess the predictive accuracy of the model and explore the estimation accuracy of the risk score model.

## Results

### Data Preprocessing and Identification of Differentially Expressed Genes

**FIGURE 1:**
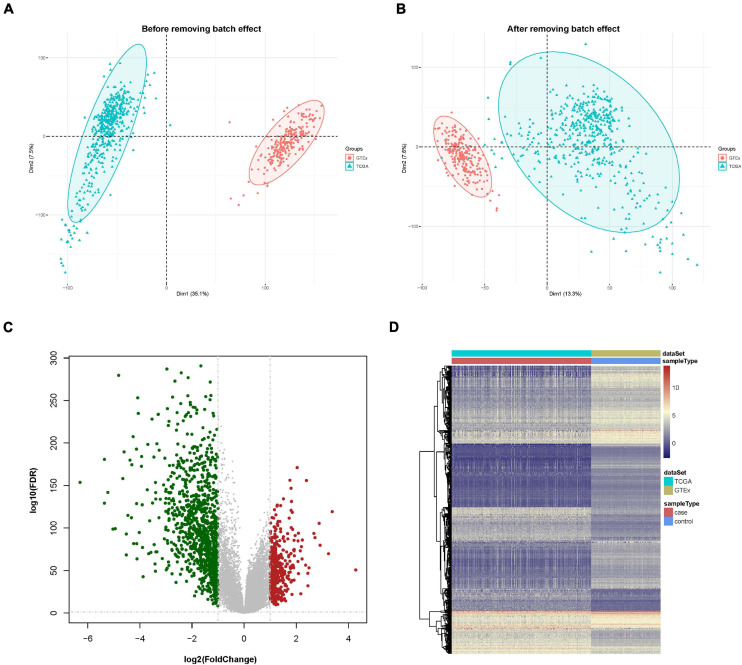
Data preprocessing and identification of differentially expressed genes (DEGs). **(A)** Principal component analysis (PCA) analysis before the removal of batch effect. **(B)** PCA analysis after the removal of batch effect. **(C)** DEG volcano plot; red dots designate upregulated genes, green dots designate downregulated genes, and gray dots designate no difference genes. **(D)** Hierarchical clustering heat map of expression values sorted according to sample (rows) and gene (columns), where the color changed from red to yellow and green suggesting gene expression changed from high to low.

First, the gene expression pattern data were integrated from two platforms (TCGA and GTEx). A total of 21,550 genes and 705 samples were obtained, including 471 cutaneous melanoma samples and 234 normal samples. Comparisons between TCGA and GTEx samples had strong batch effects (Figure 1A). We removed the batch effect and made the PCA (principal component analysis) with the PCA function in FactoMineR. A PCA plot illustrated that the batch effect between the two cohorts were well removed (Figure 1B).

A differential expression analysis was performed after removing the batch effect. Based on the threshold of log2FC > 1.0 along with FDR < 0.05, a total of 1,410 genes, consisting of 412 upregulated and 998 downregulated genes were uncovered as DEGs in contrast with the normal sample group. The DEGs are shown in a volcano map and a heat map (Figures 1C,D).

### Identification of Ferroptosis-Related Differentially Expressed Genes

**FIGURE 2:**
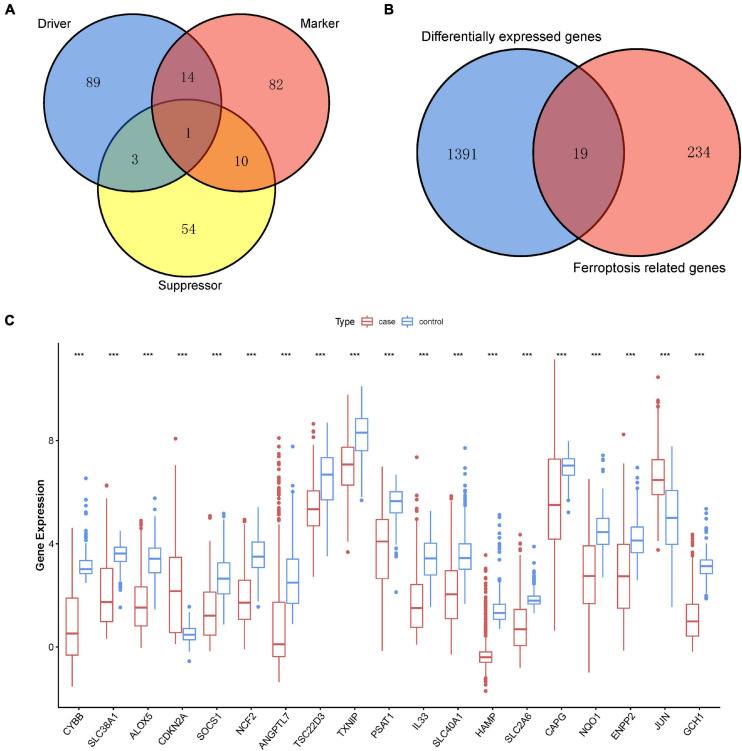
Identification of ferroptosis-related DEGs. **(A)** A Venn diagram showed ferroptosis-linked genes consisting of drivers, markers, and suppressors. **(B)** A Venn diagram illustrating that 19 ferroptosis-linked DEGs were uncovered in The Cancer Genome Atlas (TCGA) and Genotype-Tissue Expression (GTEx) Project data sets. **(C)** Box plot representing the gene expression indexes of the ferroptosis-related DEGs; red for tumor group, and blue for normal group; the X-axis showed the 19 ferroptosis-related genes, and the Y-axis represented the expression level of genes. Wilcox test was employed to assess the gene expression values between the tumor and normal groups. *p* < 0.05 signified statistical significance; *p < 0.05; ** *p* < 0.01; ***p < 0.001.

Ferroptosis-linked genes were obtained by abstracting data from FerrDb. Genes in FerrDb were annotated as markers, drivers, and suppressors. Drivers positively regulated ferroptosis, whereas suppressors negatively regulated ferroptosis. Markers indicated the occurrence of ferroptosis without regulating ferroptosis (Zhou and Bao, 2020). We obtained 253 ferroptosis-related genes including107 drivers, 68 suppressors, and 107 markers after removing genes without HGNC ID in FerrDb database (Figure 2A). After intersection ferroptosis-linked genes with DEGs, a total of 19 ferroptosis-linked DEGs were determined, of which two were upregulated genes, whereas 17 were downregulated genes (Figure 2B). Box plots showed the expression patterns of 19 ferroptosis-linked genes were expressed differentially between cutaneous melanoma and non-malignant samples (Figure 2C).

### Establishment of the Ferroptosis-Linked Gene Prognostic Model

**FIGURE 3:**
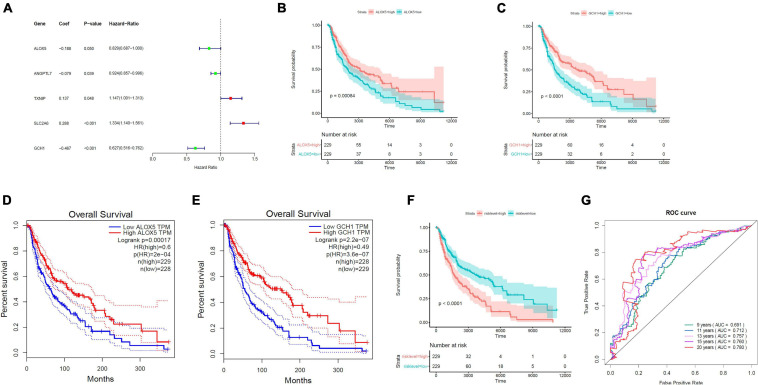
Establishment of the ferroptosis-linked gene predictive model. **(A)** The forest plots of hazard ratios showed the risk and protective factors; protective factors are indicated in green, and risk factors are indicated in red. **(B,C)** Kaplan–Meier survival curves of ALOX5 and GCH1 in the prognosis of melanoma patients. Patients with low ALOX5 and GCH1 expression exhibited a shorter overall survival rate (ALOX5, *p* = 0.00084; GCH1, *p* < 0.0001). **(D,E)** Overall survival analysis in high and low ALOX5 and GCH1 expression samples in the GEPIA data resource. **(F)** Kaplan–Meier survival curve of TCGA samples stratified into high- and low-risk groups in the entire set (*p* < 0.0001). **(G)** The ROC curves for 9-, 11-, 13-, 15-, and 20-year overall survival predictions.

On the basis of the multivariable Cox proportional hazard regression that was based on 19 ferroptosis-linked DEGs, we obtained the risk core containing the expression of five genes with the following formulas: RiskScore = −0.18811 × ALOX5 − 0.07911 × ANGPTL7 + 0.13678 × TXNIP + 0.28800 × SLC2A6 − 0.46713 × GCH1. TXNIP and SLC2A6 were risk factors, while ALOX5, ANGPTL7, and GCH1 were protective factors (Figure 3A). Kaplan–Meier curves indicated that ALOX5 (Figure 3B) and GCH1 (Figure 3C) were, respectively, served as independent prognostic factors. Survival analysis through GEPIA (Gene Expression Profiling Interactive Analysis) also verified that high ALOX5 (Figure 3D) or GCH1 (Figure 3E) mRNA expression group had a better survival outcome. Tumor samples, 458, from TCGA were stratified into high- and low-risk groups by the risk scores determined by the prediction model. The results illustrated that those patients with higher risk scores had a dismal survival time (Figure 3F). The AUC values for the ROC curve were 0.691, 0.712, 0.757, 0.760, and 0.780 for 9, 11, 13, 15, and 20 years, respectively, illustrating that the model was of good predictive ability (Figure 3G).

### Functional Assessment

**FIGURE 4:**
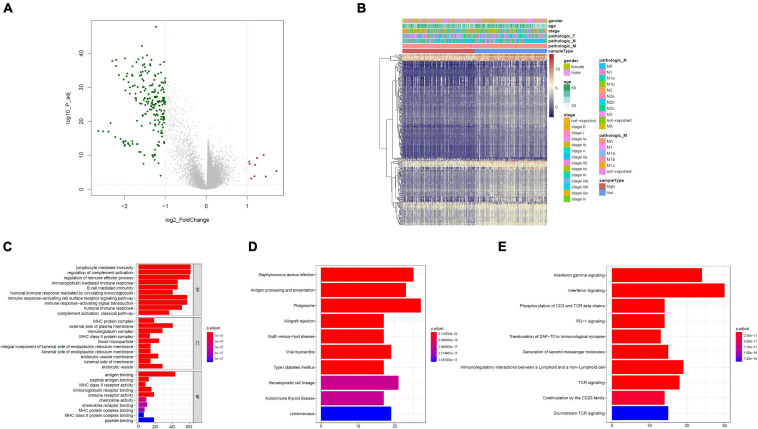
Functional assessment. **(A)** Volcano plot illustrating DEGs between the high- and low-risk groups; red dots designate upregulated genes, green dots designate downregulated genes, and gray dots designate no difference genes. **(B)** Heat map of DEGs between high- and low-risk group. **(C)** Bar plot of Gene Ontology (GO): biological process (BP), GO: cellular component (CC), and GO: molecular function (MF). **(D)** The top 10 enriched KEGG pathway terms. **(E)** The top 10 enriched reactome cascade terms. The X-axis indicates the number of rich genes, and the Y-axis indicates the pathway. Bar colors represent *p*-values.

Based on the limma package, the high-risk group had 211 downregulated genes and nine upregulated genes in contrast with the low-risk group with cutoff criteria of log2FC > 1, as well as FDR < 0.05, as illustrated in the volcano plot along with the heat map (Figures 4A,B).

To further understand the underlying biological functions, we performed GO, KEGG, and reactome analyses on the 220 DEGs. A suite of enriched functional categories related with immunity were observed in GO terms of the BP (biological process), CC (cellular component), and MF (molecular function) (Figure 4C). For the BP, “lymphocyte-mediated immunity,” “modulation of complement activation,” and “modulation of immune effector process” were the dominant terms. For the CC, “MHC protein complex,” “external side of plasma membrane,” as well as “immunoglobulin complex” were the dominant terms, and in the part of the MF section, “antigen binding,” “peptide antigen binding,” along with “MHC class II receptor activity” were the most represented terms. The KEGG database contains pathway maps that represent molecular interactions and reaction networks. A group of enriched KEGG pathways was related to immunity including “antigen processing and presentation” and “phagosome” (Figure 4D). In addition, reactome pathway analysis indicated enrichment of immune-related pathways, including “interferon gamma signaling,” “interferon signaling,” “phosphorylation of CD3 and TCR zeta chains,” “PD-1 signaling,” “translocation of ZAP-70 to immunological synapse,” and other pathways (Figure 4E). Therefore, immune-related cascades were remarkably enriched between the high- and low-risk groups.

### Immune Infiltration Analysis

**FIGURE 5:**
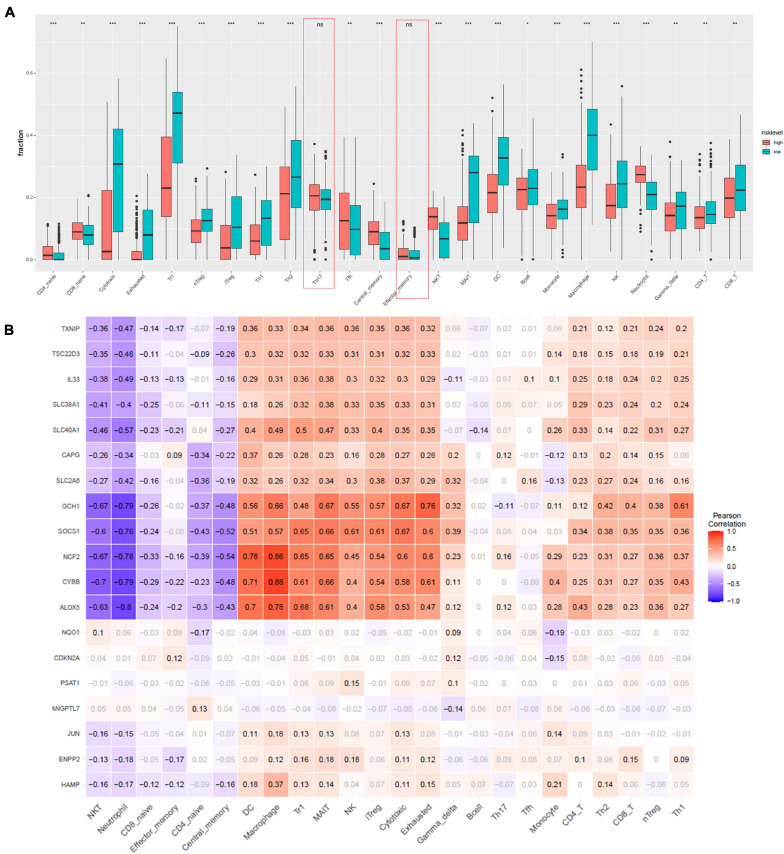
Immune infiltration analysis. **(A)** Comparisons between immune cells in the high- and low-risk groups in TCGA. *p < 0.05, **p < 0.01, ***p < 0.001, and ns designates not significant. **(B)** Correlation between the relative abundances of the 24 immune cells and the 19 ferroptosis-linked DEGs. The values in the squares represent the predicted Pearson correlation coefficients (–1 to 1.0), with black text indicating significant correlations (FDR < 0.05) and gray text. The colors of the squares denote the nature of the correlation, with Pearson’s correlation *r* = 1 for a perfect positive association, and *r* = –1 for a perfect negative relationship.

Furthermore, to characterize the immune microenvironment, we used ImmuCellAI to determine the relative abundances of 24 kinds of immune cells on the basis of the gene expression patterns of 458 samples from TCGA data resource. Infiltration of most cell types, including induced regulatory T (iTreg), CD4 T cells, natural killer T (NKT) cells, CD4 naive, cytotoxic T cells, CD8 naive, exhausted T cells, B cells, natural regulatory T (nTreg), Th1, Th2, Tfh cells, neutrophil, type 1 regulatory T cells (Tr1), central memory T cells, mucosal-associated invariant T cells (MAIT), DC, monocytes, macrophages, natural killer cells (NK), gamma delta T cells, and CD8 T cells, were remarkably different in the high- and low-risk group (Figure 5A). Only Th17 and effector memory T cells were not remarkably different between the two groups. We employed Pearson correlation analysis to validate the relationship of the 19 ferroptosis-related DEGs with the 24 immune cells. We established that the levels of expression of most ferroptosis-linked DEGs were highly linked to the abundances of multiple immune cells (Figure 5B). For example, GCH1 was remarkably positive linked to exhausted T cells, cytotoxic T cells, and MAIT, and negatively correlated with neutrophil and NKT cells. ALOX5 had a significantly positive correlation with macrophages and DCs, and negatively correlated with neutrophil and NKT cells.

### Consensus Clustering Identifies Four Subtypes of Cutaneous Melanoma

**FIGURE 6:**
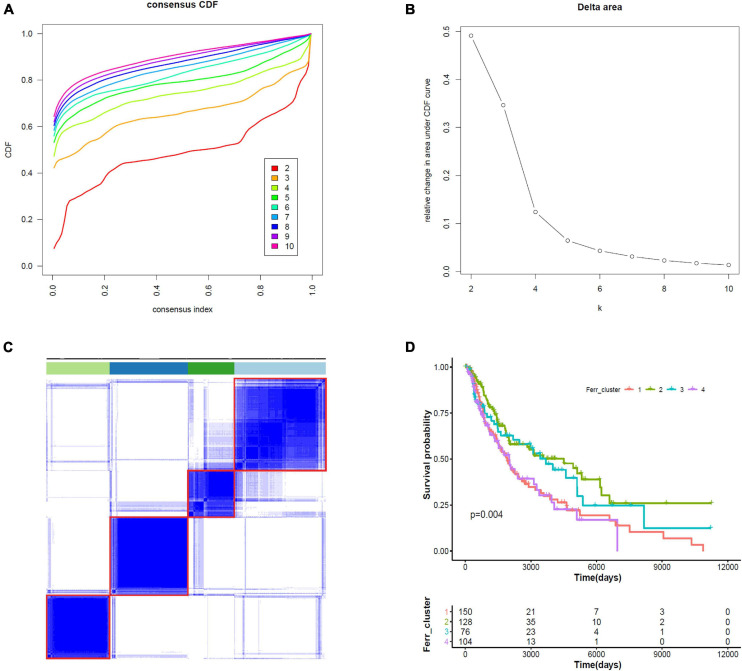
Consensus clustering identifies four subtypes of cutaneous melanoma. **(A)** Consensus clustering cumulative distribution function (CDF) from *k* = 2 to *k* = 10. **(B)** Relative change in area under the CDF curve from *k* = 2 to *k* = 10. **(C)** Consensus clustering matrix for *k* = 4. **(D)** Kaplan–Meier curves for melanoma patients stratified by four clusters.

We did consensus clustering for cutaneous melanoma samples based on 19 ferroptosis-related DEGs. Samples were clustered with the *k* value at the inflection point, which is *k* = 4, according to the elbow method (Figures 6A–C). The Kaplan–Meier assessment revealed that the survival of patients in clusters 1 and 4 were worse than that in clusters 2 and 3 (Figure 6D). These results might provide an efficient classification by ferroptosis-related DEGs.

### Validation of the Prognostic Model

**FIGURE 7:**
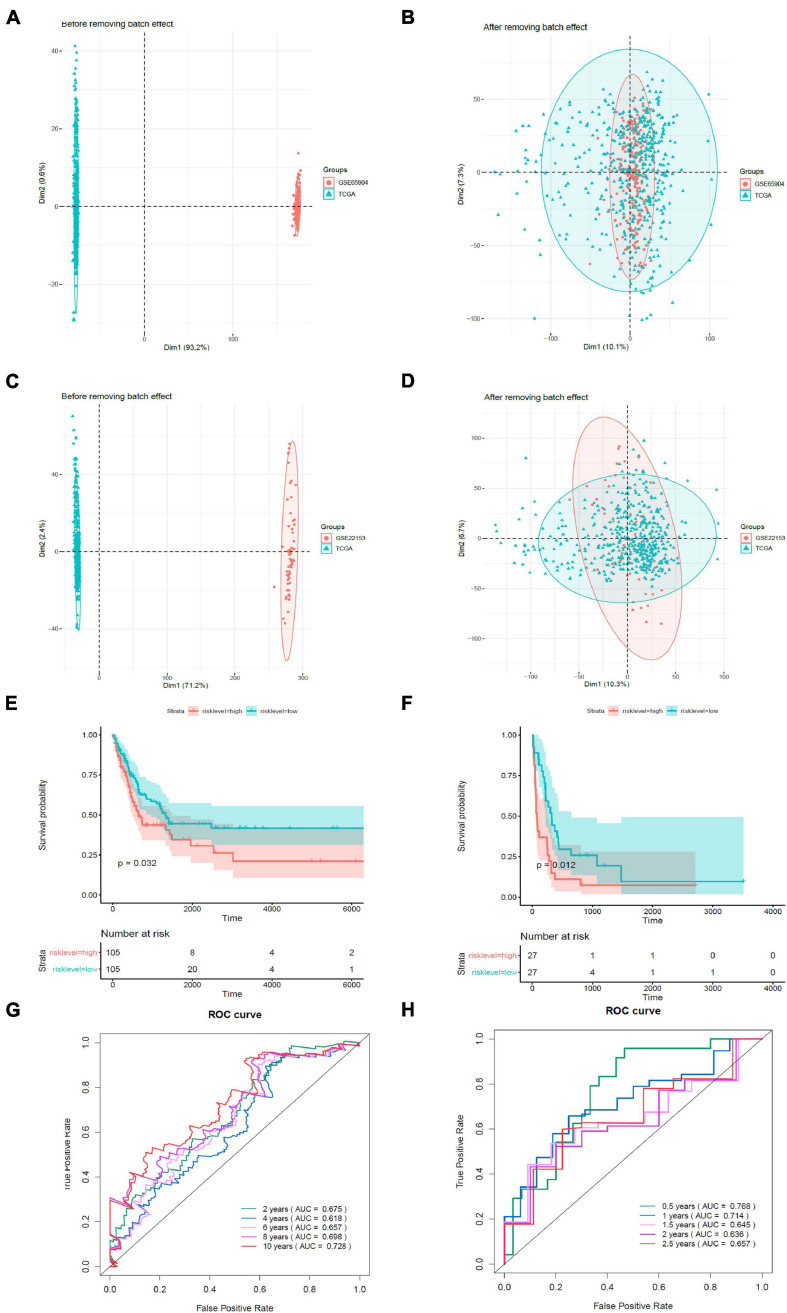
Validation of the prognostic model. **(A)** PCA analysis before removal of the batch effect between the GSE65904 dataset and the de-batched TCGA data. **(B)** PCA analysis after removal of the batch effect between the GSE65904 dataset and the de-batched TCGA data. **(C)** PCA analysis before the removal of batch effect between the GSE22153 dataset and the de-batched TCGA data. **(D)** PCA analysis after removing the batch effect between the GSE22153 dataset and the de-batched TCGA data. **(E)** Kaplan–Meier survival curve of GSE65904 dataset. **(F)** Kaplan–Meier survival curve of GSE22153 dataset. **(G)** Time-dependent ROC curves in GSE65904. **(H)** Time-dependent ROC curves in GSE22153.

We validated the estimation performance of the prognostic model using the GSE65904 and GSE22153 datasets. Comparable data were obtained after removing the batch effects (Figures 7A–D). The overall survival rates in the high-risk groups were poorer in contrast with those in the low-risk groups, both in the GSE65904 (Figure 7E) and GSE22153 (Figure 7F) datasets (*p* < 0.05). In the GSE65904 dataset, the AUC values of 2, 4, 6, 8, and 10 years were 0.675, 0.618, 0.657, 0.698, and 0.728, respectively (Figure 7G). In the GSE22153 dataset, the AUC values of 0.5, 1, 1.5, 2, and 2.5 years were 0.768, 0.714, 0.645, 0.636, and 0.657, respectively (Figure 7H).

### Validation of the Genes by qRT-PCR and Western Blot

**FIGURE 8:**
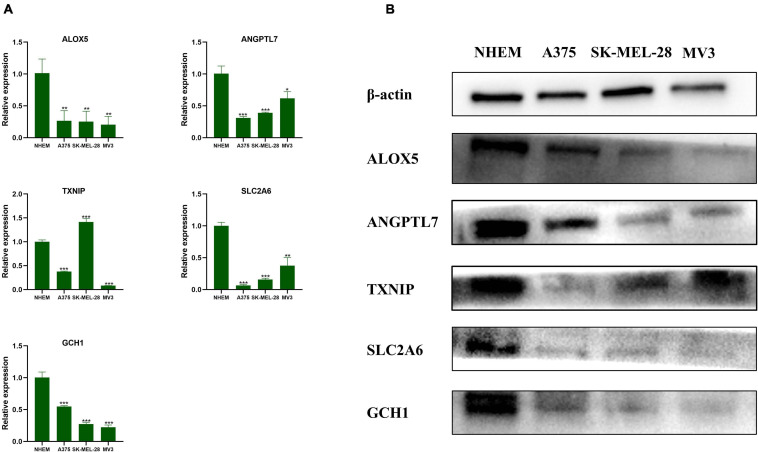
Validation of the genes by qRT-PCR and Western blot. **(A)** The relative mRNAs expression levels of ALOX5, ANGPTL7, TXNIP, SLC2A6, and GCH1 were presented by RT-qPCR. Data are shown as mean ± standard deviations, *n* = 3; *p < 0.05; **p < 0.01; ***p < 0.001. **(B)** The relative protein expression levels of ALOX5, ANGPTL7, TXNIP, SLC2A6, and GCH1 are presented by Western blot.

After verification with qRT-PCR (Figure 8A) and Western blot (Figure 8B), we confirmed that ALOX5, ANGPTL7, SLC2A6, and GCH1 were downregulated in A375, SK-MEL-28, and MV3 compared with NHEM. The relative protein expression levels of TXNIP were downregulated in all three melanoma cell lines, and the mRNAs expression levels of TXNIP were downregulated in A375 and MV3 cell lines, compared with NHEM. The results had statistical significance (*p* < 0.05).

## Discussion

In the current study, 19 ferroptosis-related DEGs were obtained in cutaneous melanoma. A novel predictive model with five ferroptosis-linked genes was first built and verified in external cohorts. Two ferroptosis-related genes were, respectively, considered as potential independent prognostic factors. Functional analysis revealed the presence of immune-related processes.

The predictive model consisted of five ferroptosis-linked genes (ALOX5, ANGPTL7, TXNIP, SLC2A6, and GCH1). ALOX5 (5-lipoxygenase) is a non-heme iron-containing dioxygenase and plays a key role in ferroptosis by inducing lipid peroxidation (Sun et al., 2019). Pharmacological repression of ALOX5 protected neurons from ferroptosis in mice with stroke (Karuppagounder et al., 2018). ALOX5 inhibition limited lipid peroxidation during ferroptosis and indirectly promoted the growth of pancreatic cancer cells (Kuang et al., 2021). Our study showed that ALOX5 may improve survival rates in melanoma by inducing ferroptosis. ANGPTL7 (angiopoietin-like protein 7) is a new pro-angiogenetic factor, which is highly expressed in colorectal, ovary, and breast cancer (Parri et al., 2014). It was reported that skin stem cells can express Angptl7 to promote lymphatic drainage (Gur-Cohen et al., 2019). Whether lymphatic drainage is related to the effect of Angptl7 on melanoma remains to be elucidated. The latest literature showed that GLO1 deletion upregulated TXNIP expression and accelerated human A375 malignant melanoma tumor growth (Jandova and Wondrak, 2021). This finding is congruent with the data of this study, in which the TXNIP was a risk factor for melanoma patients. We found that TXNIP was upregulated in the SK-MEL-28 cell line at mRNA level and downregulated at protein level compared with NHEM. It may be related to the posttranscriptional regulatory mechanism. SLC2A6 (solute carrier family 2 member 6) is a lysosomal transporter, which is modulated by NF-κB cascade (Maedera et al., 2019). GCH1 (GTP cyclohydrolase 1) is the rate-limiting enzyme in the biosynthesis of tetrahydrobiopterin (BH4) (Pickert et al., 2013). GCH1 expression level determined ferroptosis sensitivity in cancer cells (Kraft et al., 2020). Increasing BH4 levels by GCH1 overexpression augmented responses of T cells, enhancing their antitumor activity (Cronin et al., 2018). The beneficial effect of GCH1 expression on melanoma patient survival may be related to the immune response. This prognostic model is reliable, and genes in the model deserve further research.

Ferroptosis and immunotherapy are both research hotspots, and the correlations between cancer immunity and ferroptosis have drawn more and more attention (Shi et al., 2019; Du and Zhang, 2020). Excessive or lack of ferroptosis is related to a growing list of physiological and pathophysiological processes, accompanied by dysregulated immune response (Chen et al., 2021). The immune checkpoint blockade therapies are newly developed immunotherapies, which function through the activation of the natural tumor-selective killing activity of T cells (Sanmamed and Chen, 2018; Stockwell and Jiang, 2019). The important function of iron in tumor development is linked to its potential to modulate both innate and acquired immune responses, particularly in T cells and macrophages (Jung et al., 2019). Previous studies have illustrated that CD8 + T cells activated by immunotherapy sensitize tumors to ferroptosis and ultimately promote immunotherapy efficacy in melanoma (Wang et al., 2019). Interferon gamma mediates CD8 + T-cell ferroptosis-inducing activity because blocking interferon gamma could eliminate this activity of T cells. Herein, the remarkable differences of lymphocyte-mediated immunity, interferon gamma signaling, and immune cell invasion between the high- and low-risk group further suggested that targeting tumor ferroptosis-linked metabolism through interferon gamma promotes the efficacy of immunotherapy. However, an article in *Nature* reported that the unique composition of the lymphatic environment may prevent melanoma cells from ferroptosis, thereby promoting metastasis (Ubellacker et al., 2020). It remains unclear how the immune system interacts with ferroptosis. Macrophages have a vital role in the modulation of iron metabolism (Shen et al., 2021). Investigations have documented that ferroptotic cancer cells were phagocytosed by macrophages *in vitro*, confirming that that ferroptotic cells communicate with the immune cells by producing “find me” signals such as oxidized lipid mediators (Friedmann et al., 2019; Kloditz and Fadeel, 2019). There was a remarkable difference in macrophage infiltration between the high- and low-risk groups. Macrophage infiltration was linked to the expression of numerous ferroptosis-linked genes including ALOX5. Previous studies have proved that ALOX5 was involved in the synthesis of leukotriene B4 (LTB4), a proinflammatory lipid mediator production acting as a phagocyte chemoattractant (Serezani et al., 2011; Afonso et al., 2012; Orr et al., 2015). We speculated that ferroptotic cells release lipid mediators such as LTB4 through ALOX5, to attract macrophages to the site of ferroptotic cells in melanoma.

Cancer therapy has entered the age of immunity and iron (Tarangelo and Dixon, 2016; Du and Zhang, 2020). FePt nanoparticles is a novel ferroptosis-inducing agent, working by producing reactive oxygen species (ROS) by the Fenton reaction. The combination treatment of oligodeoxynucleotides containing cytosine–guanine and systemic checkpoint blockade abolishes tumors and offers a strong immunological memory effect (Zhang et al., 2019). Ferroptosis-driven nanotherapeutics combined with immunomodulation is a promising cancer treatment (Shan et al., 2020). Immunotherapy synergizing with radiotherapy can induce ferroptosis and T-cell immunity in tumor (Lang et al., 2019). By limiting immunity and ferroptosis, TYRO3 can induce anti-PD-1/PD-L1 therapy resistance in tumors (Jiang et al., 2021). A critical molecule relationship for bridging ferroptosis and immunotherapy was found to identify eligible patients for the ferroptosis-induction therapy combined with immunotherapy in clear cell renal carcinoma (Mou et al., 2021). These studies confirmed the pivotal role of ferroptosis in immunotherapy. We reason that genes in our model may guide the ferroptosis and immune combination treatment.

Although this is the first study that provides a new predictive model of five ferroptosis-linked genes in cutaneous melanoma, it still possessed some limitations that warrant consideration. First, the accuracy and applicability of the model and key prognostic genes should be validated using more prospective real-world data. Second, the underlying specific mechanisms between ferroptosis-linked genes and tumor immunity in cutaneous melanoma remained poorly understood and needed to be verified by further experimental and clinical studies.

## Data Availability Statement

The original contributions presented in the study are included in the article/Supplementary Material, further inquiries can be directed to the corresponding author/s.

## Author Contributions

CX conducted data analysis and wrote the manuscript. HC provided ideas and revised the manuscript. Both authors contributed to the article and approved the submitted version.

## Conflict of Interest

The authors declare that the research was conducted in the absence of any commercial or financial relationships that could be construed as a potential conflict of interest.

## Publisher’s Note

All claims expressed in this article are solely those of the authors and do not necessarily represent those of their affiliated organizations, or those of the publisher, the editors and the reviewers. Any product that may be evaluated in this article, or claim that may be made by its manufacturer, is not guaranteed or endorsed by the publisher.

## Footnotes

1
http://www.genome.jp/kegg/

2
http://www.reactome.org

3
https://CRAN.R-project.org/package=survminer

4
https://CRAN.R-project.org/package=survivalROC
